# Three-Dimensional Study of *F. graminearum* Colonisation of Stored Wheat: Post-Harvest Growth Patterns, Dry Matter Losses and Mycotoxin Contamination

**DOI:** 10.3390/microorganisms8081170

**Published:** 2020-08-01

**Authors:** Xavier Portell, Carol Verheecke-Vaessen, Rosa Torrelles-Ràfales, Angel Medina, Wilfred Otten, Naresh Magan, Esther García-Cela

**Affiliations:** 1 School of Water, Energy and Environment, Cranfield University, Cranfield, Bedfordshire MK43 0AL, UK; xavier.portell@cranfield.ac.uk (X.P.); wilfred.otten@cranfield.ac.uk (W.O.); 2 Applied Mycology Group, School of Water, Energy and Environment, Cranfield University, Cranfield, Bedfordshire MK43 0AL, UK; c.verheecke@cranfield.ac.uk (C.V.-V.); rosaatorrelles@gmail.com (R.T.-R.); a.medinavaya@cranfield.ac.uk (A.M.); n.magan@cranfield.ac.uk (N.M.); 3 School of Life and Medical Sciences, University of Hertfordshire, Hatfield, Hertfordshire AL10 9AB, UK

**Keywords:** Cereals, silo, fungi, modelling, 3D colonisation, respiration, ergosterol, zearalenone, deoxynivalenol, trichothecenes.

## Abstract

*Fusarium* causes significant post-harvest quality losses and mycotoxin contamination in stored wheat but the colonisation dynamics of the grain and how this may be affected by the initial inoculum position in the grain mass is poorly understood. This study examined the 3D growth kinetics and mycotoxin production (deoxynivalenol and zearalenone) by *F. graminearum* during hyphal colonisation from different initial inoculum positions in wheat microcosms (top-centre, bottom-centre, and bottom-side) maintained at two water activities (a_w_; 0.95 and 0.97). Clear jars were used to visually follow the colonisation dynamics. Fungal respiration and associated dry matter loss (DML) and ergosterol were also quantified. Colonisation dynamics was shown to be affected by the inoculation position. At the end of the colonisation process, fungal respiration and DML were driven by the inoculation position, and the latter also by the prevailing a_w_. Fungal biomass (ergosterol) was mainly affected by the a_w_. The initial inoculum position did not affect the relative mycotoxin production. There was a positive correlation between respiration and ergosterol, and between mycotoxin production and colonisation indicators. We suggest that spatially explicit predictive models can be used to better understand the colonisation patterns and mycotoxin contamination of stored cereal commodities and to aid more effective post-harvest management.

## 1. Introduction

Wheat is susceptible to *Fusarium* head blight (FHB) world-wide which causes both yield and quality losses, in addition to contamination of harvested grain with mycotoxins, particularly zearalenone (ZEN) and type B trichothecenes such as deoxynivalenol (DON). There are strict regulations in place for maximum contamination levels of these two toxins to reduce accumulation in the human and animal food chains. Both mycotoxins can be produced pre- and post-harvest. Although *Fusarium graminearum* requires higher moisture content levels compared to other more xerotolerant or xerophilic spoilage fungi (e.g., *Penicillium* and *Aspergillus* species, respectively), delayed or inefficient drying and unsafe storage practices can allow fungal growth and potential mycotoxin accumulation.

Recent studies have shown that under conducive moisture contents and over a wide range of temperatures, *F. graminearum* colonisation of stored wheat grain can cause significant dry matter losses (DMLs) and result in DON and ZEN contamination exceeding the EU maximum limits for unprocessed cereals for human food consumption (DON: 1.750 and ZEN: 100 µgKg^−1^) [1] and animal feed (DON: 8.000 and ZEN: 2.000 µgKg^−1^) [2]. Overall, <1% DML can result in significant impacts on quality losses and toxin contamination exceeding the EU limits [3,4,5].

Stored cereals in silos represent an ecosystem in which there are significant interactions between the abiotic and biotic factors [6]. Indeed, Limay-Rios et al. [7] showed that the main sources for contamination of stored wheat in grain silos are at the top of the silo, where the inlet for grain filling is placed, and the outlet, where the grain is removed from the silo. These areas and the silo walls represent areas where higher moisture content can occur due to moisture migration, leading to increased contamination with mycotoxins.

However, there is very little information on how fungal growth is initiated and the way in which mycelial colonisation occurs, especially in three dimensions. Most previous studies have been conducted by measuring colonisation in two directions or dimensions only [8,9]. However, in reality, filamentous fungi such as *F. graminearum* grow in stored grain in three dimensions extending their mycelium in all directions to obtain nutrients from the rich wheat substrate. Du et al. [10] and Vidal-Diez de Ulzurrun et al. [11] recently reported novel lattice-based models for a three dimensional study of fungal growth, but there are no publications that have examined *Fusarium* colonisation in stored cereal grain.

Measurement of ergosterol, fungal biomass and colony diameter have been positively correlated on agar media previously suggesting that the three parameters may be useful for primary modelling [12,13,14]. Colony diameter is extensively used to assess fungal growth. However, in aged colonies (>30 days) ergosterol did not increase exponentially, suggesting that despite the colony expansion in terms of area, there was a decrease in the rate of ergosterol accumulation [12]. Similarly, fungal diameter measurements may not be a perfect indicator of fungal biomass, especially in aging colonies where biomass may increase despite limitation in actual colony extension [13]. Other studies have used ergosterol content as a measure of the overall mould contamination levels of commodities and related this to safe or unsafe storage conditions [15,16]. In addition, the use of ergosterol was found to be an effective biochemical marker for early indications of fungal contamination as well as of predicted DON contamination levels in stored cereals [17,18].

However, few studies, if any, have examined the colonisation of cereals by a mycotoxigenic species in three dimensions to try to understand the growth kinetics and the relationship between growth and toxin production. There is also a lack of knowledge of how temporal fungal colonisation occurs in a 3D matrix such as stored grains via the intergranular spaces, and how this may be affected by the initial inoculum position or the prevailing environmental factors.

The objectives of this study were to examine the kinetics of 3D spatial colonisation of stored wheat grain by *F. graminearum* and the accumulation of DON and ZEN when inoculated in different positions and under different water availabilities in laboratory microcosm storage systems. This allowed information to be obtained on (a) spatial patterns of colonisation depending on the different initial inoculum positions, (b) the changes in respiration and associated DMLs, (c) quantification of fungal biomass, using ergosterol, (d) the temporal DON and ZEN mycotoxin accumulation during colonisation and (e) the correlation between these different biological and physical parameters.

## 2. Materials and Methods

### 2.1. Fungal Isolate and Spores Preparation

*F. graminearum* strain Fg 08/111 isolated from wheat in the UK was cultivated on V8 vegetable juice plates (V8^®^, 175 mL; CaCO_3_, 3 g; ZnSO_4_·7H_2_O, 0.01g; CuSO_4_·5H_2_O, 0.005g; agar, 20 g·L^−1^) and incubated in the dark at 25 °C for ten days to obtain heavily sporulating cultures [19]. Following incubation, 3 mL of sterile Tween 80^®^ (0.05%) was poured on the Petri plates previously incubated and then gently scratched with a sterile Drigalsky’s spatula. The liquid was transferred to a second Petri plate containing fungi and the procedure repeated with the remaining 4 Petri plates. The resulting liquid was recovered and collected in a sterile falcon tube. After homogenizing, the number of spores per mL was determined using a Thoma counting chamber (Celeromics, Grenoble, France) and the final concentration adjusted to 1 × 10^4^ spores·mL^−1^ in sterile water + Tween 80 (0.005%).

### 2.2. Grain Preparation, Inoculation and Incubation

Dry wheat grain (0.71 water activity (a_w_), 13.5 % moisture content (m.c.)) was irradiated with 12–15 kGy (Synergy Health, Swindon, UK) to reduce microbial contaminants while maintaining germinative capacity of the grain, and stored at 4 °C until use. The grain a_w_ levels were modified to 0.95 and 0.97 by adding known amounts of sterile water and reference to an adsorption curve for this batch of wheat grain, which was detailed in Garcia-Cela et al. [20], and verified using the Aqualab 4TE a_w_ meter.

Square jars of 136 mL capacity (Pattersons Glass Ltd, Grimsby, UK) (see supplementary Figure S1) with a 9 mm diameter hole in the jar lid, which was closed with a cotton wool plug to allow gaseous exchange while avoiding contamination, were autoclaved at 121 °C for 20 min. Single grains inoculated with a drop of 5 µL of the 10^4^ spores mL^−1^ suspension (approx. 50 spores per grain) were placed in distinct positions to provide initial inoculum of *F. graminearum* for initiating growth in the different positions within the jars: (a) top-centre, (b) bottom-side and (c) bottom-centre. For the bottom inoculations, an 8 µL drop of organic clear nail polish was used to fix the grain on the jar bottom. Fixed grains were inoculated with the stated spore solution drop and 1 min later, the remaining grains were added to the jar. For the top-centre position, the spore solution drop was placed over a single grain in the centre of the top layer of grains. In all cases, 15 jars per treatment combination were used, including 3 samples without *F. graminearum* inoculated per a_w_ tested.

Inoculated jars were incubated at 25 °C for 10 days. To maintain atmospheric equilibrium relative humidity, jars were placed in food storage containers (10 L volume) containing beakers of glycerol–water solution at the treatment a_w_ level (6 × 100 mL per container). Glycerol–water solutions were renewed once (day 5) during the experimental period.

### 2.3. Colonisation Pattern Assessment

Fungal colonisation of the wheat grain was followed by recording the superficial mycelial extension visible through the six sides of the square clear jars (supplementary Figure S2). Clear labels with a mesh of 0.5 × 0.5 cm were aligned and pasted on the bottom and sides of the jars. To avoid external contamination of the grains, jar lids were opened under sterile conditions and a plastic sheet with the same mesh was aligned on the top. Visual fungal colonisation was followed every 24 h until the grain was fully colonised.

The colonisation data were used to compute volumetric colonisation (cm^3^) by *F. graminearum* from the three inoculation treatment positions. In two dimensional studies, fungal growth is known to expand in circular colonies following a radial pattern [21]. In the present study, we expanded the approach assuming that mycelial colonisation followed an ellipsoidal shape before being constrained by the cubic shape of the jar. The detailed procedure followed to compute the volumetric colonisation is presented in Appendix A.

### 2.4. Respiration Determination and Dry Matter Loss Estimation

Every 24 h, samples were sealed for 1 h at 25 °C with injection lids that contained polytetrafluoroethylene (PTFE)/silicone septa (20 mm diameter × 3.175 mm thickness) to allow for CO_2_ accumulation in the headspace. Headspace volumes of jars containing grain at 0.95 or 0.97 a_w_ were, respectively, 90.67 and 88.33 mL. In both growth conditions, 5 mL of headspace air were withdrawn with a syringe and directly inserted into the Gas Chromatograph (GC) for CO_2_ analysis. An Agilent 6890N Network GC (Agilent Technologies, Cheshire, UK) with a Thermal Conductivity Detector and helium as a carrier gas was used. A Chromosorb 103 packed column was usedand the data were analysed using Agilent Chemstation Software (Agilent Technologies, Cheshire, UK).

GC was calibrated with a standard gas bottle of 10.18% CO_2_ and 2% O_2_ in nitrogen (British Oxygen Company, Guilford, Surrey, UK). *F. graminearum* CO_2_ production was obtained by subtracting the sample respiration of a blank culture that had not been inoculated with the fungal species. The percentages of CO_2_ production were used to calculate the Respiration (R) in mg CO_2_ (kg·h^−1^), total cumulative production CO_2_ and total DML as described in Mylona and Magan [3].

### 2.5. Mycotoxins Extraction and Analysis

Before mycotoxin extraction, samples were dried at 60 °C for 24 h and then milled in a laboratory blender (Waring Commercial, Christian, UK). Mycotoxins accumulation of three replicate samples per combination of inoculation position and a_w_ on alternate days (days 2, 4, 6, 8 and 10) were extracted by adding 500 µL of acetonitrile:water:formic acid (79:20.9:0.1, *v*:*v*:*v*) to 100 (± 10) mg of milled wheat and agitated for 90 min at 300 rpm at 25 °C on a rotary shaker (miniShaker VWR, Leighton Buzzard, UK). Then, samples were centrifuged for 10 min at 22,600 g (Centrifuge 5417S Eppendorf, Stevenage, UK), and 1 or 3 µL of the supernatant was injected into an Exion LC series HPLC linked to a 6500+ qTRAP-MS system in Electrospray Ionisation (ESI) mode (Sciex Technologies, Warrington, UK). An ACE 3-C18 column (2.1 × 100 mm, 3 µm particle size; Hichrom) with guard cartridge (4 × 3 mm, Gemini, Agilent) was conditioned at 40 °C. The elution gradient used water:acetic acid (*v*:*v*, 99:1, solvent A) and methanol:acetic acid (*v*:*v*, 99:1) (solvent B), both supplemented with 5 mM ammonium acetate. The 15 min-long gradient included: 0 min, 90% A; 0–2.0 min, 90–60% A; 2.0–10.0 min, 0% A and 10.0–11.50 min, 0% A; 11.50−12.0 min, 90% A; 12.00−15.0 min, 90% A at a 0.3 mL·min^−1^ flow rate. 10 msec of dwell time per daughter ion (2 per metabolites) was used in an unscheduled Multiple Reaction Monitoring (MRM) in both positive and negative mode using the parameters listed in Table 1.

The source conditions used included: Curtain gas 40%, Collision Gas Medium, IonSpray voltage 4500 V/ 5500 V, Temperature 400 °C, both ion sources gas at 60 psi, with a 10 V Entrance Potential for all compounds. Data acquisition was conducted with Analys^t®^ Data Acquisition version 1.6.3, and quantification through MultiQuant™ version 3.0.3.

The analyte recovery, the limit of detection (LOD) and the limit of quantification (LOQ) were calculated using recommendations stated elsewhere [22]. The calculated recoveries were used as correction factors to calculate the absolute concentration of the analytes. The LODs were 0.26 and 2.29 ng·g^−1^ and the LOQs 0.85 and 7.63 ng·g^−1^ for ZEN and DON, respectively.

### 2.6. Ergosterol Analysis

The replicates and treatments were oven-dried at 60 °C for 48 h, milled in a laboratory blender (Waring Commercial, Christian, UK), homogenised, and the ergosterol content analysed using an adaptation of the rapid ultrasonic method developed previously [23]. This involved adding 2 mL of deionised water to a 5 g of sample. After 15 min, 10 mL methanol:ethanol (*v*:*v*, 4:1) was added and samples were stored at 4 °C for 2 h. Then, 20 mL hexane:propan-2-ol (*v*:*v*, 98:2) was added to each sample and they were ultrasonicated at 150 W in an ice bath with water for 3 min 20 s. After 30 s, 2 mL of the supernatant was centrifuged at 7000× *g* for 10 min, from which 1.5 mL was used for HPLC analysis.

The HPLC system was a Shimadzu Class-VP, which consisted of SCL-10Avp controller, SPD-10Avp detector and LC-10ADvp pump (Shimadzu Corporation, Japan). An amount of 100 µL was injected into a Lichrosorb Phenomenex column (250 × 4.6 mm, 10 µm) at 35 °C. Run time for samples was 15 min with ergosterol being detected at about 9.20 min. The flow rate of the mobile phase (hexane:propan-2-ol; *v*:*v*, 98:2) was 1.4 mL·min^−1^.

Calibration standards of 5, 25, 50, 100 and 150 µg·g^−1^ ergosterol in hexane:propan-2-ol (*v*:*v*, 98:2) were used as an external calibration. A general recovery test was conducted from spiked samples using 4 replicates. The procedure was the same as explained above, with two changes: (a) 1 mL of internal standard was added after the deionised water and samples were allowed to stand for 15 min, (b) 19 mL of hexane:propan-2-ol (*v*:*v*, 98:2) was added instead of 20 mL. Average recovery rates for 50, 100, 500 and 1000 µg·g^−1^ ergosterol from wheat grain matrix were 89.18 ± 14.44%, 104.35 ± 12.37%, 106.37 ± 9.81% and 94.41 ± 5.97%, respectively.

### 2.7. Data Analysis

Data were analysed with JMP Pro (JMP®, version 14. SAS Institute, Inc., Cary, NC, USA). All data were assessed for normality and homoscedasticity. Growth rate data, DML, respiration and ergosterol, were normally distributed, homoscedastic and independent. Therefore, data sets were analysed using two-way Analysis of Variance (ANOVA) for the determination of the significance between a_w_ treatments and inoculation position. Then, Student′s t- or Tukey′s HSD test were used to identify significant differences between groups. Mycotoxins accumulation data were not normally distributed, therefore a Kruskal–Wallis test for the determination of the significance between a_w_ treatments and inoculation position was undertaken. Then, non-parametric comparisons for each pair was conducted using the Wilcoxon test. The statistical relationship between variables was assessed by Pearson or Spearman correlations. A signification level of 5% was assumed for all statistical analysis.

The “fit_growthmodel” function of the “growthrates” R package [24] was used to fit the colonisation data to a two-phase linear model of the form:

(1) $$C_{t}=\left(t>\lambda \right)*R_{C}*\left(t-\lambda \right),$$

where *C_t_* is the colonisation (cm^2^ or cm^3^) at time *t* (days), *λ* a lag phase with 0 colonisation (days) and *R_c_* the colonisation rate (cm^2^·day^−1^ or cm^3^·day^−1^).

## 3. Results

### 3.1. Fungal Colonisation

**Figure 1:**
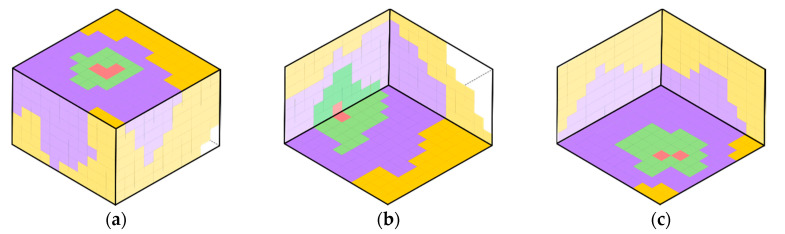
Example of the visual observation of the fungal colonisation dynamics at 0.95 a_w_ starting from different inoculation positions: (**a**) top-centre, (**b**) bottom-side and (**c**) bottom-centre. In the figure, colours depict colonisation after day 2 (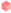), day 3 (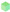), day 4 (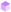), day 5 (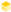) and day 6 (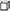).

**Figure 2:**
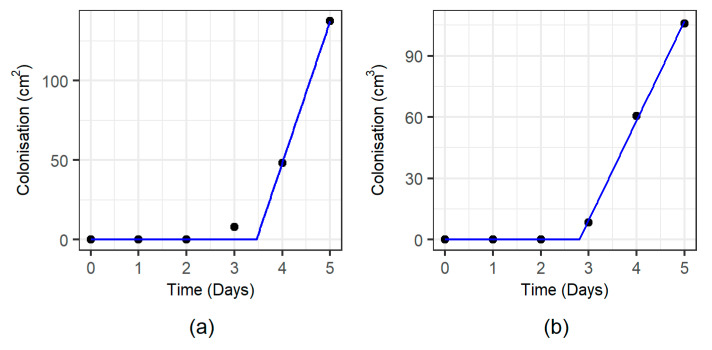
Example of the two-phase linear model fit (blue line) for the colonisation data (dots) recorded for the sample grown on 0.95 a_w_, bottom-centre inoculation, second replication. (**a**) Model fit to surface colonisation data, and (**b**) model fit to volume colonisation data.

**Figure 3:**
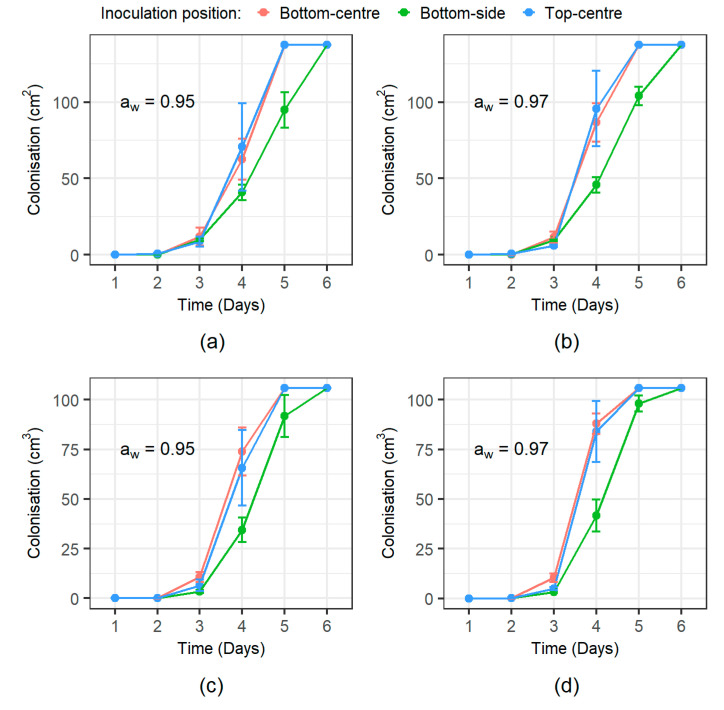
Temporal colonisation of the wheat grain by *F. graminearum* in both a_w_ treatments from the three different initial inoculum positions. (**a**) Superficial colonisation for 0.95 a_w_ treatment, (**b**) superficial colonisation for 0.97 a_w_ treatment, (**c**) volumetric colonisation for 0.95 a_w_ treatment, and (**d**) volumetric colonisation for 0.97 a_w_ treatment. Bars show standard deviation of the three replicates.

*F. graminearum* showed a rapid colonisation rate at both tested a_w_ levels with all of the wheat grain visually completely colonised after 5–6 days. A comparative example of the recorded superficial growth showed distinct colonisation patterns of *F. graminearum* depending on the initial inoculum position (Figure 1). Superficial colonisation data were well described using a two-phase linear model (Figure 2) providing estimates of the lag time prior to colonisation and the superficial colonisation rate (cm^2^·day^−1^) shown in Table 2. The lag phase length prior to superficial colonisation was shown to be significantly affected by the inoculation position but not by a_w_, with mean lag phase times higher for the bottom-side position (2.97 ± 0.22 h) than the bottom-centre (2.92 ± 0.31 h) and top-centre (2.94 ± 0.32 h) positions. No significant differences (α = 0.05) in the colonisation rate were detected among treatments. As a consequence of this increased lag time, the bottom-side inoculations were able to completely colonise the grain surface only by day 6 of the experiment. For the other inoculum positions (top-centre and bottom-centre) this occurred after 5 days (Figure 3a,b).

Volumetric fungal colonisation (cm^3^) was obtained from the visible colonisation assuming that the fungal growth of the grain was consistent with a partial ellipsoidal expansion of the mycelia constrained by the cubic volume of the jar (see Appendix A). Volumetric colonisation (Figure 3b,c) was also well adjusted to a simple two-phase linear model, obtaining colonisation lag times and volumetric colonisation rate (cm^3^·day^−1^) estimates shown in Table 2. The length of the lag phase was shown to be significantly affected by the inoculation position (*p*-value = 0.003), with the bottom-side inoculation having an extended lag period (3.28 ± 0.16 h) distinguishable from that of the top-centre (2.89 ± 0.21 h) and bottom-centre (2.76 ± 0.14 h) inoculum positions. The volumetric colonisation rate were significantly affected by a_w_, and was more rapid at 0.97 a_w_ (69.52 ± 15.24 cm^3^/day) than at 0.95 a_w_ (51.10 ± 7.42 cm^3^·day^−1^).

### 3.2. Indirect Indicators of Fungal Growth

#### 3.2.1. Fungal Respiration Dynamics

**Figure 4:**
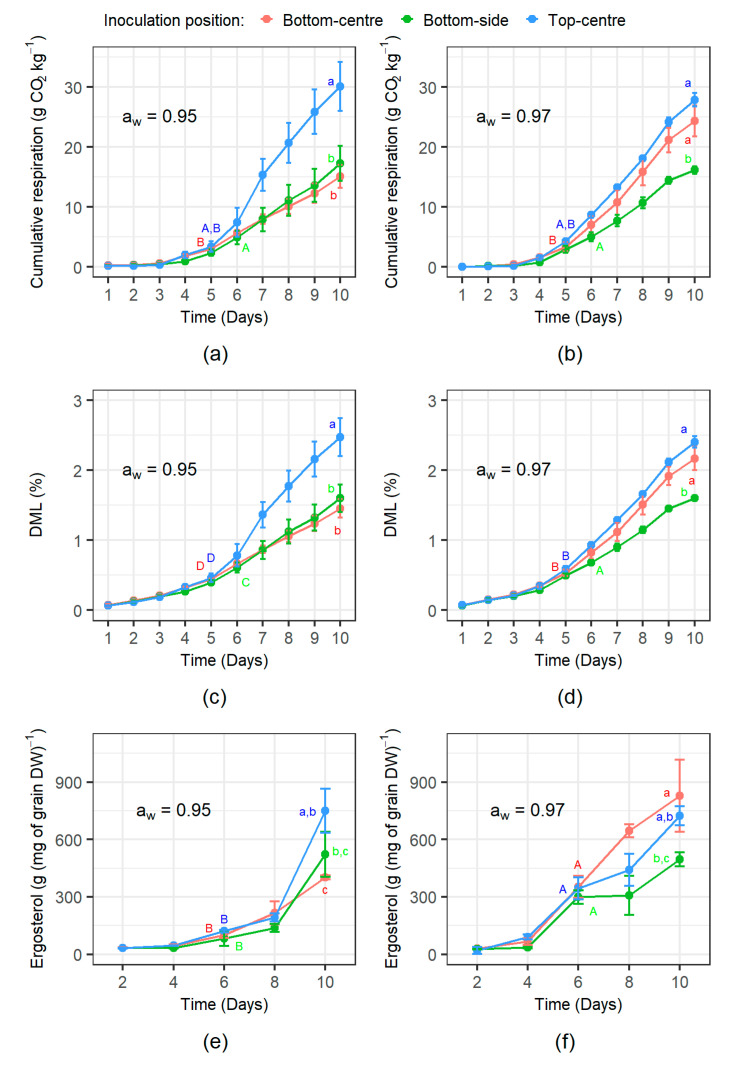
Temporal evolution of the indirect indicators of fungal growth from three different inoculum positions. Cumulative respiration of *F. graminearum* at (**a**) 0.95 a_w_ and (**b**) 0.97 a_w_. The dry matter loss (DML; %) of wheat grain inoculated with *F. graminearum* at (**c**) 0.95 a_w_ and (**d**) 0.97 a_w_. Ergosterol content of *F. graminearum* inoculated grain at (**e**) 0.95 a_w_, and (**f**) 0.97 a_w_. Bars show standard deviation of the three replicates. Upper case and lower case letters show statistically significant differences among treatments at the end of the colonisation and at the end of the experiment, respectively.

Cumulative fungal respiration for the 10 day experimental period is shown in Figure 4a,b. In all inoculation positions and a_w_ treatments, respiration increased over time as the *F. graminearum* mycelia colonised the wheat grain. The accumulated respiration at the end of the colonisation process (days 6 for bottom-side, and day 5 for top-centre and bottom centre inoculations) was significantly affected by the initial inoculation position (*p*-value = 0.022). Fungal colonisation from the bottom-side proceeded more slowly but produced more CO_2_ (4.96 ± 0.84 g·CO_2_·kg^−1^) than the top-centre (3.83 ± 0.79 g·CO_2_·kg^−1^) and bottom-centre (3.23 ± 0.52 g·CO_2_·kg^−1^). Bottom-side respiration was significantly higher (α = 0.05) than the bottom-centre respiration. However, these two treatments were indistinguishable from the top-centre cumulative respiration.

The ANOVA analysis at the end of the experiment (day 10), suggested a significant effect of the inoculation treatment position (*p*-value < 0.001) and an interaction amongst the inoculation positions and a_w_ factors (*p*-value = 0.003). The 0.95 a_w_ top-centre, the 0.97 a_w_ top-centre, and 0.97 a_w_ bottom-centre treatments were similar. The other treatments: 0.95 a_w_ bottom-side, 0.97 a_w_ bottom-side, and 0.95 a_w_ bottom-centre, were similar but significantly lower than the previous group (Figure 4a,b).

#### 3.2.2. Dry Matter Loss Dynamics

The DML followed a similar temporal increase at both a_w_ levels depending on the inoculum position (Figure 4c,d) and reflected the respiration rates (see Figure 4a,b). However, there was a more linear increase earlier than that for the respiration rate. At the end of the colonisation period (days 6 for bottom-side, and day 5 for top-centre and bottom-centre inoculations) the DML was significantly affected by a_w_ levels (*p*-value = 0.001) and the inoculation position (*p*-value = 0.002). The DMLs were higher at 0.97 a_w_ than in the slightly drier 0.95 a_w_ treatment (0.60 ± 0.07 vs. 0.51 ± 0.09% respectively). As observed for respiration, grain colonisation initiated from the bottom-side position proceeded more slowly and generated more DML (0.64 ± 0.06%) by the time the grain was completely colonised than the top-centre (0.53 ± 0.08%) and bottom-centre (0.49 ± 0.06%) treatments.

The ANOVA analysis at the end of the experiment (day 10) suggested a significant effect of both a_w_ (*p*-value = 0.018) and inoculation position (*p*-value < 0.0001) treatments, and for the interaction between these two factors (*p*-value = 0.003). The 0.95 a_w_ top-centre, 0.97 a_w_ top-centre and 0.97 a_w_ bottom-centre treatments were very similar. The remaining treatments again grouped together and had significantly lower DMLs than the other group of treatments.

#### 3.2.3. Ergosterol Production Dynamics

During the colonisation process, ergosterol content of all of the treatment combinations appeared to be mainly driven by the storage a_w_ (Figure 4e,f). By day 6, close to the end of the colonisation of the wheat grain, a_w_ had a significant effect (*p*-value < 0.001) on ergosterol content, although was interestingly not influenced by the inoculation position (*p*-value = 0.07). Overall, there was a significantly higher ergosterol content at 0.97 a_w_ (331.56 ± 50.76 g mg^−1^) than at 0.95 a_w_ (100.97 ± 27 g·mg^−1^). At the end of the experimental period, ergosterol content was statistically affected by the a_w_ (*p*-value = 0.027), inoculation position (*p*-value = 0.005) and the interaction between these two factors (*p*-value = 0.004). The 0.97 a_w_ bottom-centre produced a higher ergosterol content than the bottom-side inoculation and 0.95 a_w_ bottom-centreinoculation. Top-centre inoculations were very similar statistically to the 0.97 a_w_ bottom-centre treatment and the bottom-side inoculations. Similarly, bottom-side inoculations could not be distinguished from the 0.95 a_w_ bottom-centre treatment.

### 3.3. Mycotoxin Production Dynamics

**Figure 5:**
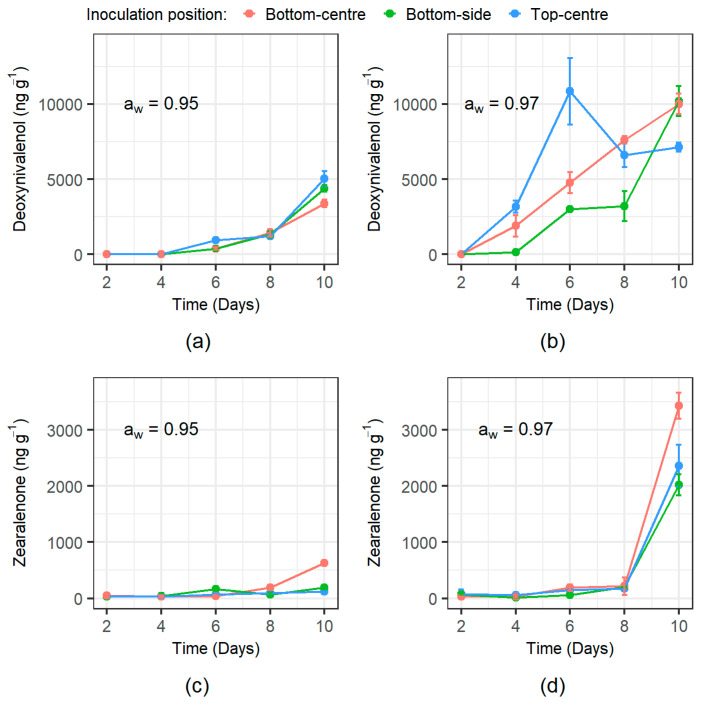
Temporal deoxynivalenol (DON) and zearalenone (ZEN) mycotoxin production from each inoculation position at the two water activity treatments examined. (**a**) DON at 0.95 a_w_; (**b**) DON at 0.97 a_w_; (**c**) ZEN at 0.95 a_w_; and (**d**) ZEN at 0.97 a_w_. Bars show the standard deviation of the mean.

Mycotoxin production was significantly impacted by the a_w_ treatment (*p*-value < 0.0001 and *p*-value = 0.0331 for DON and ZEN, respectively) with higher production occurring at 0.97 a_w_. DON production could be quantified from day 4 for the wetter 0.97 a_w_ (Figure 5b) condition and from day 6 in the drier 0.95 a_w_ treatment (Figure 5a). For ZEN production, this was predominantly found from day 6 onwards in both a_w_ treatments (Figure 5). ZEN increased markedly by the end of the experimental period (10 days). Unlike for colonisation of the wheat grain, the inoculum position did not significantly impact either DON or ZEN production.

### 3.4. Correlation between Experimental Measures

#### 3.4.1. Correlation between Fungal Respiration and Ergosterol Content

There was a significant correlation between ergosterol content and cumulative respiration data for *F. graminearum* inoculation in wheat grain. Spearman rank order correlation suggested a strong positive correlation between these two variables (Table 3). When considering individual a_w_ treatments the correlation was stronger during the colonisation period but this decreased when both a_w_ levels are considered together. The overall correlation coefficient among water activities for the length of the experiment was 88.58%.

#### 3.4.2. Correlation between Mycotoxin Contamination Levels and Indicators of Colonisation

Correlations between the growth indicators used in this study and the mycotoxin production is shown in Table 4. For DON, all of the indicators used (i.e., volume, ergosterol and cumulative DML) showed a positive correlation. The correlations ranged from 0.8149 to 0.9528 and were dependent on the a_w_ treatment, with higher correlations at 0.95 a_w_ than 0.97 a_w_. The cumulative DML and the ergosterol content were found to be the indicators most correlated to DON contamination level.

For ZEN, all indicators except for the volume of growth obtained at 0.95 a_w_ correlated well with the mycotoxin content. The average correlation ranged from 0.4150 to 0.8193. Higher correlation factors were observed at 0.97 a_w_ when compared to 0.95 a_w_. The best correlated indicators for ZEN were the cumulative DML at 0.97 a_w_ and both cumulative DML and Ergosterol at 0.95 a_w_.

Overall, DON and ZEN production showed a good correlation at 0.95 a_w_ (0.6417) and 0.97 a_w_ (0.7604).

## 4. Discussion

This study found differences in the colonisation patterns from different initial inoculum positions for a mycotoxigenic filamentous fungus such as *F. graminearum*. The inoculation position affected the length of the lag phase but not the actual rates of mycelial colonisation. This suggests that the geometry of the mycelia expanding through the rich nutritional matrix had an effect on the fungal expansion via the intergranular spaces. To our knowledge, this is the first attempt to follow and model the volumetric colonisation of such stored commodities. An assumption was made that colonisation followed an ellipsoidal front. Volumetric data obtained was adjusted assuming a two phase linear model. Being a non-linear transformation, one would expect the non-constrained volumetric colonisation to be better fitted by a non-linear relationship. Nonetheless, in our conditions, this could be explained by the fact that the colonisation was constrained by the finite cubic shape of the storage jars shortly after initial growth. Alternative techniques for examining the volume of colonised grains could include visualisation techniques that allow exploration of internal fungal growth, such as resin impregnation and thin slicing [25], or non-invasive Computed Tomography (CT). Visualisation of the fungal colonisation using X-ray CT is currently challenging due to the similar absorption properties of fungi and organic matter, although it may be possible for fungi accumulating minerals and colonisation in wood [26].

Unlike the superficial colonisation rate, volumetric colonisation rates were significantly different between a_w_ levels. Significant differences in the volumetric colonisation pattern between a_w_ treatments were found in this study that followed similar (pseudo) 2D fungal growth patterns where mycelial colonisation by *F. graminearum* of layers of wheat grain was more rapid in wetter grain [8,9]. However, the differences in colonisation patterns in the former studies only examined superficial colonisation rates and did not take account of a volumetric 3D grain matrix with multiple grain layers as opposed to a single layer of grain. The exploratory nature of this filamentous fungus is thus able to more effectively utilise the nutrients from the wheat grain in different directions.

Analysis of the cumulated respiration at the end of the colonisation period suggested that (i) fungi required more energy to colonise the grain when the colonisation was slower, and (ii) that the a_w_ levels tested do not significantly affect this process. Differences in the grain colonisation dynamics from different initial inoculum positions may be related to the ability of the hyphae to grow into the intergranular spaces to colonise other wheat grains and produce the necessary extracellular enzymes to exploit the rich nutritional substrate effectively. This may have resulted in the differences observed in respiration dynamics with faster colonisation (bottom-centre and top-centre) than other inoculum positions. The low respiration levels found in the bottom-centre colonisation at 0.95 a_w_ may thus have been due to lower oxygen levels in the intergranular spaces and the more stressful water availability treatment. Overall, the present results show that fungal colonisation rates can be accurately estimated by the amount of CO_2_ produced in different stored cereals [3,5,27,28].

In the present study, fungal respiration rates and DML obtained by CO_2_ measurements supported the colonisation data sets. Fungal respiration rates have been correlated with DML, as fungal growth produces CO_2_ due to the oxidation of carbohydrates and production of water vapour and heat during aerobic respiration [29]. DML has been previously used as a quality indicator of stored grains [29,30]. Therefore, the differences among contamination points reported in the present study suggests that contamination source might have an impact on quality losses in a storage silo.

In our study, ergosterol as a fungal biomass measurement was shown to be affected by both a_w_ level and the inoculation position. Considering that the analysis performed at day 6 did not allow the comparison of the ergosterol amounts at the exact end of the colonisation, the statistical analyses showed the effect of inoculum position on the fungal biomass produced based on ergosterol. This supports the hypothesis that the fungal biomass composition changes depending on the rates of colonisation. A higher ergosterol production in the wetter conditions (0.97 a_w_) was probably due to the increased utilisation of the carbohydrates in the wheat grain by the *F. graminearum.* Overall, the present results are similar to the ergosterol levels found in wheat cultivars infected by *F. graminearum* by Stuper-Szablewska et al. [31], although they used a different ergosterol analysis method. Despite the absence of a widely accepted analysis method for ergosterol as a biomass indicator in cereals, it has been directly correlated with fungal colonisation of cereals and DON contamination [18,32,33]. However, as it is a destructive method it cannot be performed in real time. Thus, few attempts have been made in food science to develop a high-recovery method for this fungal biomass indicator in food commodities [34,35,36].

This study showed that respiration rates and ergosterol content have a highly significant positive correlation. Previously, DML has been successfully correlated with *Fusarium* mycotoxin levels [3,5] and can be calculated from measured respiration rates. Therefore, the correlation between respiration rate and ergosterol found suggests that further research should be conducted to examine the relationship between ergosterol content, respiration rates and mycotoxin contamination levels.

Finally, mycotoxin production (both DON and ZEN) was found to be unaffected by the inoculation position but was higher in the wetter growth condition. The higher DON and ZEN production at 0.97 a_w_ when compared to 0.95 a_w_ was due to the effect of the relative water stress the mycotoxigenic species was exposed to. *F. graminearum* is more sensitive to drier conditions [9]. The present study showed earlier production of DON at day 4 at 0.97 a_w_ compared to day 6 at 0.95 a_w_. Our results also showed ZEN production starting at day 6 independently of the a_w_ tested and an increase in production by day 10. Our study also showed that production of these mycotoxins occurred within 6 days, with the inoculum position having no effect on relative DON and ZEN production patterns. Previously, strains of *F. graminearum* from Argentina were shown to produce higher amounts of DON at 0.97 a_w_ (43 ng·g^−1^) compared to none at 0.95 a_w_ in wheat gains after 7 days incubation [8]. They also found that at 0.95 a_w_ production of DON only occurred after 14 days. Ezekiel et al. [37] monitored the production of ZEN in wheat at 0.95 a_w_ every 6 days and showed that production only occurred after day 12 followed by a steady increase in ZEN production.

The lack of effect of the inoculation position in mycotoxin content may be related to the fact that the whole sample was homogenized before the mycotoxin extraction. One previous report showed larger toxin clusters and stronger spatial autocorrelation in the outer grain layers in a silo, in which the higher humidity and more favourable oxygen availability resulted in better fungal development, while the presence of toxins in deeper locations in the stored grain was related to the influence of gravity [38]. This highlights that more information is needed about the differences between outer and inner layers within a large grain mass in terms of fungal growth and toxin contamination.

This study showed a strong correlation between cumulative DML and DON production found at both a_w_ levels (0.9220 at 0.95 a_w_; 0.8669 at 0.97 a_w_), similar to that of Mylona et al. [4] who found a 0.9572 spearman correlation between DML and DON production on wheat at three different a_w_ levels (0.89, 0.94, 0.97 a_w_) at 15 to 30 °C. The present study also showed a significant correlation between ZEN production and DML (0.5971 at 0.95 a_w_; 0.8193 at 0.97 a_w_). Similar correlations were observed by Garcia-Cela et al. [5] and Mylona et al. [4]. Both correlations for DON and ZEN production provide effective information to develop post-harvest management tools to be integrated for improved Decision Support Systems (DSS).

## 5. Conclusions

In this study, the behaviour of *F. graminearum* in stored wheat in terms of grain colonisation and mycotoxin production (DON and ZEN) was evaluated in a 3D volume for a period of ten days. Primary inoculum position affected the initial growth significantly and therefore the colonised grain. Modern silos are currently monitored at different spatial levels, consequently, spatial modelling could be used to predict the level of risk. Respiration and DML indicators seem to be as reliable as ergosterol measurements (to indicate fungal colonisation) but with the advantage that they can be monitored in real-time. Thus, to perform efficient silo management, different approaches must be tailored to each of the spatial areas covered by the sensors in which the alert was detected.

The results of this study revealed that understanding the fungal growth pattern and the diffusion of multiple mycotoxins is essential for the development of accurate predictive models that can support effective post-harvest management of grain. This is critical as grain is traded on a wet weight basis and very slight changes in the moisture can lead to an increase in the activity of mycotoxigenic spoilage moulds and mycotoxin contamination.

## Figures and Tables

**Table 1:** Overview of the metabolite studies in the mycotoxin analysis. The first Q3 for each was used for quantification. DP: Declustering potential, CE: Collision Energy, CXP: Cell Exit potential.

Analyte	Retention Time (min)	Q1 (m/z)	DP (V)	Q3 (m/z)	CE (V)	CXP (V)
3-AcetylDeoxynivalenol	3.86	397.3	−70	−59.2/−307.1	−38/−20	−8/−7
15-AcetylDeoxynivalenol	3.84	339.1	91	137.2/321.2	17/13	8/18
Deoxynivalenol	2.60	355.1	−70	−59.2/−265.2	−40/−22	−13/−10
Zearalenone	7.33	317.1	−110	−175/−121.1	−34/−42	−13/−8

**Table 2:** Superficial and volumetric colonisation rates. Lag times and colonisation rates were obtained by adjusting the data to a two-phase linear model.

Water Activity (a_w_)	Inoculation	Replicate	Superficial Colonisation		Volumetric Colonisation	
			Lag Time (Days)	Rate (cm^2^·Day^−1^)	Lag Time (Days)	Rate (cm^3^·Day^−1^)
0.95	Top-centre	1	2.71	59.50	2.66	48.93
0.95	Top-centre	2	3.46	45.30	2.75	48.76
0.95	Top-centre	3	2.90	64.25	3.25	60.67
0.95	Bottom-side	1	3.35	51.25	3.18	39.08
0.95	Bottom-side	2	3.04	47.50	3.40	59.93
0.95	Bottom-side	3	2.79	43.67	3.33	59.80
0.95	Bottom-centre	1	2.69	63.63	2.54	46.22
0.95	Bottom-centre	2	2.88	63.50	2.80	48.73
0.95	Bottom-centre	3	3.51	92.50	2.65	47.78
0.97	Top-centre	1	2.82	63.75	2.94	94.35
0.97	Top-centre	2	2.61	60.87	2.83	50.91
0.97	Top-centre	3	2.83	64.50	2.93	78.37
0.97	Bottom-side	1	2.85	43.93	3.27	54.07
0.97	Bottom-side	2	2.75	44.15	3.01	51.18
0.97	Bottom-side	3	3.04	47.88	3.46	64.12
0.97	Bottom-centre	1	2.65	65.50	2.87	75.23
0.97	Bottom-centre	2	2.92	66.38	2.84	81.10
0.97	Bottom-centre	3	2.79	65.62	2.89	76.33

**Table 3:** Spearman rank order correlation coefficient between cumulative fungal respiration and ergosterol content. *p*-values of all coefficients were < 0.001.

Water Activity Levels	0.95 a_w_	0.97 a_w_	0.95 + 0.97 a_w_
Days 2, 4 and 6	0.9193	0.9329	0.7796
Days 2, 4, 6, 8 and 10	0.8785	0.9404	0.8858

**Table 4:** Correlation (Spearman’s) factors of the mycotoxin content with volumetric colonisation, ergosterol, and Cumulative dry matter loss. DON: Deoxynivalenol, ZEN: Zearalenone, DML: dry matter loss. Correlation to the volume was performed using data from the days 2, 4 and 6 of growth, time when the samples were fully colonised.

Mycotoxins	Fungal Growth Indicator	0.95 a_w_		0.97 a_w_	
		Correlation	*p*-Value	Correlation	*p*-Value
DON (ng/g)	Volume (cm^3^)	0.8149	<0.0001	0.8528	<0.0001
	Ergosterol (mg·g^−1^)	0.9528	<0.0001	0.8972	<0.0001
	Cumulative DML	0.9220	<0.0001	0.8660	<0.0001
ZEN (ng/g)	Volume (cm^3^)	0.4150	0.0313	0.7490	<0.0001
	Ergosterol (mg·g^−1^)	0.6686	<0.0001	0.7856	<0.0001
	Cumulative DML	0.5971	<0.0001	0.8193	<0.0001
DON (ng/g)	ZEN (ng·g^−1^)	0.6417	<0.0001	0.7604	<0.0001

**Figure A1:**
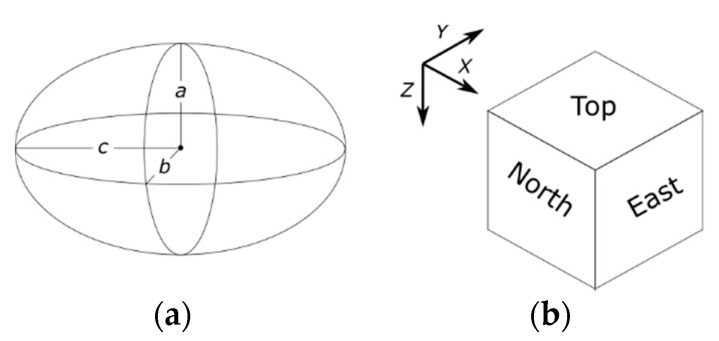
Radii and spatial configuration assumed in the study. (**a**) Ellipsoid showing radii of the fungal colonisation, and (**b**) coordinate system and faces of the medium.

## Supplementary Materials

The following are available online at https://www.mdpi.com/2076-2607/8/8/1170/s1, Figure S1: Clear square jars used in this experiment, Figure S2: Labelling system used to follow 3D colonisation of wheat grains by *F. graminearum*.

## Appendix A. Computation of the Grain Colonised Volume

Three-dimensional fungal colonisation was assumed to follow an ellipsoidal shape of radii *a*, *b* and *c* (Figure A1). After the initial stages of colonisation, the ellipsoidal expansion was constrained by the finite volume of the jar and a number of assumptions were required. The procedure followed for the centre and side positions is described below.

### Appendix A.1. Top-Centre and Bottom-Centre Positions

Volume computation could be generalized in two main cases:

1. Mycelial expansion just observable on the top (bottom) side. Volumetric colonisation at the time *t* ($V_{t}$) was computed assuming a half ellipsoidal shape (Equation (A1))
  (A1) $$V_{t}=\frac{2}{3}\pi \cdot a\cdot b\cdot c,$$
  where *a*, *b* and *c* are elliptic radii. Radii *b* and *c* where computed as the maximum minus the minimum coordinates showing colonisation along X and Y axis divided by two, and *a* was assumed to be the $0.75\cdot \frac{b+c}{2}$. This assumes an upwards (downwards) radial reduction due to the fact that the mycelial expansion proceeds through a more tortuous path that the surface as observed experimentally [39].
2. Mycelial expansion was observable on the vertical sides of the grain volume. Colonised volume was approximated as cubic shape and a half ellipsoid (Equation (A2)) as follows:
  (A2) $$V_{t}=L_{\hat{X}}\cdot L_{\hat{Y}}\cdot L_{\hat{Z}}+\frac{2}{3}\pi \cdot \frac{L_{\hat{X}}}{2}\cdot \frac{L_{\hat{Y}}}{2}\cdot a,$$
  where $L_{\hat{X}}$, $L_{\hat{Y}}$ and $L_{\hat{Z}}$ are mean distances obtained along axes X, Y and Z, respectively, showing colonisation. Radii *a* (in cm) was computed as 3.5 − $L_{\hat{Z}}$ if the face opposite to the inoculation point (bottom and top, respectively, for top-centre and bottom-centre inoculations, respectively) showed any sign of colonisation, or as the minimum value between $0.75\cdot \;\frac{L_{\hat{X}}-L_{\hat{Y}}}{2}$ and 3.5 − $L_{\hat{Z}}$ if not.

### Appendix A.2. Bottom-Side Position

Fungi was inoculated at the bottom-side position of the jar. The vertical face of the jar where the fungi was inoculated was denoted as North face, their adjacent faces as East and West, and the opposed vertical face denoted South. Colonisation patterns fell into three main cases:

1. First mycelial expansion not observable on either the East or West faces. The colonisation was assumed to follow a quarter of ellipsoid (Equation (A3)):
  (A3) $$V_{t}=\frac{1}{3}\pi \cdot a\cdot b\cdot c,$$
  where *a*, *b* and *c* are elliptic radii. Radius *a* was obtained on the North face as the mean value of the colonisation shown along the Z-axis. Radius *c* was obtained on the North face as the maximum minus the minimum coordinates showing colonisation along the X-axis divided by two. Radius *b* was obtained on the bottom face as the mean value of the colonisation shown along the Y-axis, assuming a minimum value of 1 square.
2. Mycelial expansion observable on either the East or West faces. The growth was assumed to follow a cylindrical shape (V_c_) and an ellipsoid shape (V_e_) as expressed in Equation (A4):
  (A4) $$V_{t}=\frac{V_{e}}{8}+\frac{V_{c}}{4}=\frac{1}{12}\pi \cdot a\cdot b\cdot \left(c-\frac{L_{W}}{2}\right)+\frac{1}{4}\pi \cdot a\cdot b\cdot \frac{L_{W}}{2},$$
  where $L_{W}$ is the wide of the jar (5.5 cm).
3. Mycelial expansion observable on the top or South faces. Colonisation front was assumed to follow a cylindrical shape until the end of the colonisation (Equation (A5)) according the expression:
  (A5) $$V_{t}=L_{W}\cdot a\cdot L_{T}+L_{W}\cdot L_{S}\cdot \left(b-L_{T}\right)+\frac{1}{4}\pi \cdot \left(b-L_{T}\right)\cdot \left(a-L_{S}\right)\cdot L_{W}$$
  where *L_T_*, and *L_N_* are the colonisation length attained by the fungi on the top and South faces, respectively. These were computed as the mean value of the colonisation shown along the Y-axis of the top face (*L_T_*) and the Z-axis of the South face (*L_S_*).
