# Supplementary material for: Three-Dimensional Study of F. graminearum Colonisation of Stored Wheat: Post-Harvest Growth Patterns, Dry Matter Losses and Mycotoxin Contamination
Source: Microorganisms. 2020 Aug 1;8(8):1170. doi: 10.3390/microorganisms8081170 (PMC7465026; doi:10.3390/microorganisms8081170)
Supplement: Supplementary file 1 [file microorganisms-08-01170-s001.pdf]

microorganisms

MDPI

Supplementary Material

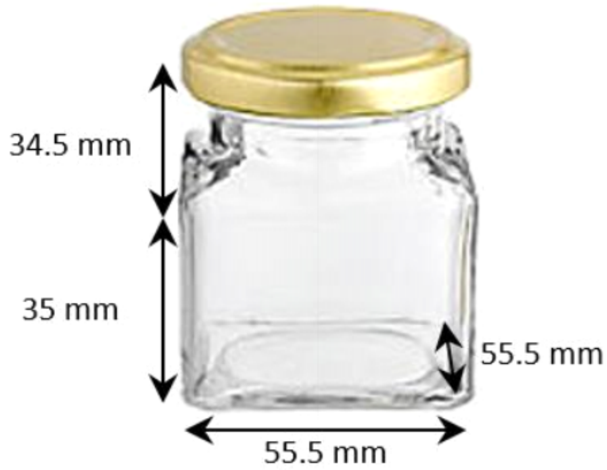

Figure S1. Clear square jars used in this experiment. Adapted from Pattesons Glass Ltd (nd).

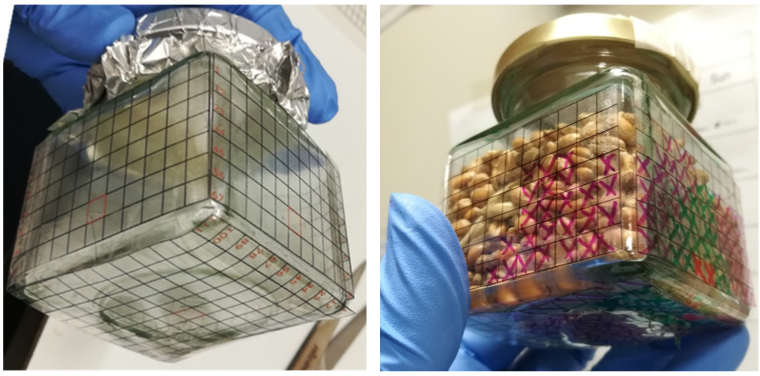

(**a**) (**b**)

Figure S2. Labelling system used to follow 3D colonisation of wheat grains by *F. graminearum*. (**a**) Jar with square mesh labels without grains, and (**b**) example of the 3D colonisation attained after 4 days of incubation.
